# Effectiveness of workplace social distancing measures in reducing influenza transmission: a systematic review

**DOI:** 10.1186/s12889-018-5446-1

**Published:** 2018-04-18

**Authors:** Faruque Ahmed, Nicole Zviedrite, Amra Uzicanin

**Affiliations:** Community Interventions for Infection Control Unit, Division of Global Migration and Quarantine, Centers for Disease Control and Prevention (CDC), Atlanta, GA USA

**Keywords:** Influenza, Distancing, Community mitigation, Non-pharmaceutical, Systematic review, Telework, Workplace

## Abstract

**Background:**

Social distancing is one of the community mitigation measures that may be recommended during influenza pandemics. Social distancing can reduce virus transmission by increasing physical distance or reducing frequency of congregation in socially dense community settings, such as schools or workplaces. We conducted a systematic review to assess the evidence that social distancing in non-healthcare workplaces reduces or slows influenza transmission.

**Methods:**

Electronic searches were conducted using MEDLINE, Embase, Scopus, Cochrane Library, PsycINFO, CINAHL, NIOSHTIC-2, and EconLit to identify studies published in English from January 1, 2000, through May 3, 2017. Data extraction was done by two reviewers independently. A narrative synthesis was performed.

**Results:**

Fifteen studies, representing 12 modeling and three epidemiological, met the eligibility criteria. The epidemiological studies showed that social distancing was associated with a reduction in influenza-like illness and seroconversion to 2009 influenza A (H1N1). However, the overall risk of bias in the epidemiological studies was serious. The modeling studies estimated that workplace social distancing measures alone produced a median reduction of 23% in the cumulative influenza attack rate in the general population. It also delayed and reduced the peak influenza attack rate. The reduction in the cumulative attack rate was more pronounced when workplace social distancing was combined with other nonpharmaceutical or pharmaceutical interventions. However, the effectiveness was estimated to decline with higher basic reproduction number values, delayed triggering of workplace social distancing, or lower compliance.

**Conclusions:**

Modeling studies support social distancing in non-healthcare workplaces, but there is a paucity of well-designed epidemiological studies.

**Systematic review registration number:**

PROSPERO registration # CRD42017065310.

**Electronic supplementary material:**

The online version of this article (10.1186/s12889-018-5446-1) contains supplementary material, which is available to authorized users.

## Background

Influenza pandemics occur when new influenza A viruses emerge that spread from person to person in an efficient and sustained way. Over the past 100 years, the clinical severity of influenza pandemics has ranged from moderate for the 2009 pandemic to very high for the 1918 pandemic [1]. The economic impact of the next influenza pandemic in the United States, in the absence of vaccination and other mitigation measures, has been estimated to be $71 to $166 billion [2]. A vaccine against a new pandemic virus might not be widely available for up to 6 months given current vaccine production technology [3]. The 2017 Update of the Pandemic Influenza Plan published by the US Department of Health and Human Services includes community mitigation measures, known also as nonpharmaceutical interventions (NPIs), that can be used before a pandemic vaccine is widely available [4]. The goals of community mitigation are to delay the influenza peak to buy time for the development and administration of a well-matched pandemic vaccine; reduce the peak number of daily influenza cases to decrease stress on the health-care system and to protect critical infrastructure (by reducing daily absenteeism rates); and reduce the overall number of influenza cases in order to decrease morbidity and mortality [3]. NPIs include personal protective measures, environmental measures, and community measures aimed at increasing social distancing. Social distancing can reduce virus transmission from infected persons to susceptible individuals by increasing physical distance between people or reducing frequency of congregation in socially dense community settings, such as schools or workplaces [3]. The US Pandemic Influenza Plan as well as the World Health Organization Public Health Research Agenda for Influenza have called for more research on the effectiveness, timing, and optimal implementation of social distancing measures in different community settings [4, 5]. Research on the effectiveness of social distancing has focused on schools, most notably on pre-emptive school closures, for which systematic reviews have been published [6, 7].

Of the US civilian noninstitutionalized population aged ≥16 years, about two-thirds participate in the labor force [8]. The influenza illness attack rate in the workplace in a severe pandemic can be over 20% [1]. Contacts made in the workplace represent 20–25% of all weekly contacts, and influenza transmission in the workplace represents on average 16% (range 9–33%) of all transmissions [9]. Social distancing measures in non-healthcare workplaces can include increased use of telecommuting and remote-meeting options, staggered work hours, and spacing workers further apart [3]. The objective of this systematic review is to assess the evidence that social distancing interventions in non-healthcare workplaces, compared to no intervention, reduce or slow influenza transmission among workers and in the general population.

## Methods

The protocol for this systematic review was registered on PROSPERO, an international prospective register of systematic reviews (ID # CRD42017065310) [10]. The systematic review was conducted following the Preferred Reporting Items for Systematic Reviews and Meta-Analysis (PRISMA) statement (see Additional file 1) [11]. The inclusion criteria included randomized controlled trials, epidemiological studies, and modeling studies reporting results of social distancing interventions in non-healthcare workplaces. The exclusion criteria included the following: review articles, commentaries, and editorials; studies in animals; studies conducted in health-care, school, or university settings; and studies on workplace closure (workplace closure is not a recommended NPI [3]). Studies on generic social distancing that did not specifically mention workplace social distancing were also excluded. The primary outcomes of interest were the following: cumulative influenza attack rate (percentage of individuals in a given population who will get influenza illness); peak influenza attack rate; time to peak; lost workdays; and harms.

### Literature search strategy and study selection

Electronic searches of the published and grey literature were conducted using MEDLINE, Embase, Scopus, Cochrane Library, PsycINFO, Cumulative Index to Nursing and Allied Health Literature (CINAHL), NIOSHTIC-2, and EconLit to identify studies published in English during the period January 1, 2000, through May 3, 2017. The search terms are provided in PROSPERO [10]. Two reviewers (FA and NZ) independently identified eligible articles by screening titles and abstracts and reviewing full-text articles. The reference lists of included studies were examined to search for additional studies.

### Data extraction and risk of bias assessments

Two reviewers (FA and NZ) extracted data independently from all included studies using a standard form that was piloted. Variables for which data were sought included the following: study dates, study design, predominant influenza strain, threshold for triggering social distancing, basic reproduction number (R_0_), population characteristics, type of intervention (including duration of intervention), comparator, type of outcome measures, setting, publication status, and funding source. Two reviewers (FA and NZ) independently assessed the quality of epidemiological studies using the Risk of Bias in Non-randomized Studies of Interventions (ROBINS-I) tool [12]. Risk of bias for each domain is classified into four categories: *low* (study is comparable to a well performed randomized trial), *moderate* (study is sound for a non-randomized study but cannot be considered comparable to a well performed randomized trial), *serious* (study has some important problems), and *critical* (study is too problematic to provide any useful evidence on the effects of intervention). A particular level of risk of bias for an individual domain means that the overall risk of bias for the study is at least this severe. Any disagreements were resolved through discussion or a third reviewer (AU). The quality of modeling studies was not assessed.

### Data synthesis

Percentage reductions were calculated using the following formula: Percentage reduction = ((Attack rate in the absence of intervention – Attack rate with intervention) /Attack rate in the absence of intervention) × 100 [6]. A narrative synthesis was performed [13].

It was decided a priori to present results by basic reproduction number (R_0_), a measure of virus transmissibility. R_0_ is defined as the average number of secondary cases produced by a typical infectious case in a fully susceptible population [14]. A reproduction number greater than 1 indicates that the infection will grow in the population, whereas a value less than 1 indicates that the infection will decline [14]. Higher R_0_ values are associated with higher cumulative attack rates [15]. Factors that affect R_0_ include the population contact rate, the probability of infection per contact, and the duration of illness. The results are presented using three R_0_ categories: ≤ 1.9; 2.0–2.4; and ≥ 2.5 [6]. The R_0_ of the 1918 influenza pandemic was somewhat higher than those of the 1957, 1968, and 2009 pandemics, but the R_0_ values of all four pandemics were estimated to be less than 1.9 [14]. The characteristics of influenza pandemics are unpredictable, and the higher R_0_ categories provide estimates for an atypical pandemic.

## Results

**Fig. 1:**
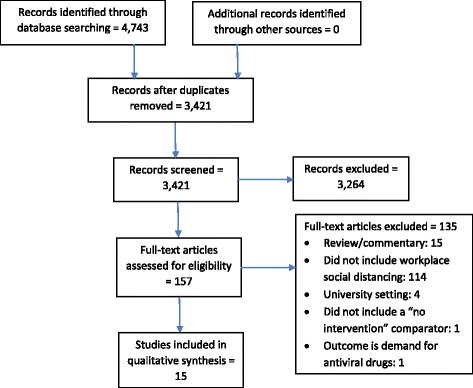
PRISMA flow diagram of study selection

The selection of eligible studies is shown in the PRISMA flow diagram (Fig. 1). The database search identified 4743 records. After removal of duplicates, 3421 records were screened. Among the excluded studies, 10 included workplace closure and one did not include a “no intervention” comparator [16–26]. Fifteen studies, representing three epidemiological [27–29] and 12 modeling [30–40], met the inclusion criteria. Nine studies were from North America, four from Asia, one from Europe, and one from Australia (Appendix). The funding sources of the studies were government (10 studies), university (2 studies), research council (1 study), industry (1 study), and none/unknown (1 study).

Social distancing measures in the epidemiological studies included segregation of persons into small subgroups and working from home (Appendix). These studies showed reductions in seroconversion to 2009 influenza A (H1N1), occurrence of influenza-like illness (ILI), and workplace attendance with severe ILI (which would result in reduced transmission) (Table 1). The overall risk of bias in the epidemiological studies was serious in two studies and critical in one study (Table 2). All three studies had moderate or serious risk of bias in the confounding domain, and two studies had moderate risk of bias in the outcome measurement domain. In addition, because the outcomes used in these studies were surrogates for influenza illness, the evidence was indirect [41].

**Table 1:** Percentage reduction in cumulative influenza attack rate, 2000–2017

First author, year published	Country	Influenza strain	Intervention^b^	Percentage reduction^a^		
				R_0_ ≤ 1.9	R_0_ = 2.0–2.4	R_0_ ≥ 2.5
Epidemiological studies^c^						
Rousculp, 2010 [27]	USA	Seasonal influenza A(H3N2), 2007–2008	Single	20	–	–
Kumar, 2012 [28]	USA	2009 influenza A(H1N1) pandemic	Single	36	–	–
Lee, 2010 [29]	Singapore	2009 influenza A(H1N1) pandemic	Multiple	61	–	–
Modeling studies						
Timpka, 2016 [30]	Sweden	Future pandemic strain	Single	12^d^	–	–
Zhang, 2012 [31]	Singapore	Not reported	Single	18	–	–
Mao, 2011 [32]	USA	Seasonal scenario (R_0_ = 1.4) and a pandemic scenario (R_0_ = 2.0)	Single	82	23	–
Xia, 2013 [33]	China	2009 influenza A(H1N1) pandemic	Single	–	–	–
			Single + VAC	–	–	–
Milne, 2008 [34]	Australia	Pandemic strain	Single	28	13	7
			Multiple	94	96	95
Milne, 2013 [35]	Papua New Guinea	Pandemic strain	Multiple	63^e^	–	–
Miller, 2008 [36]	USA	Influenza A(H3N2) in population with no prior immunity	Multiple	88	–	–
Andradottir, 2011 [37]	Canada	2009 influenza A(H1N1) pandemic	Multiple	30	–	–
			Multiple + VAC	61	–	–
			Multiple + AV	73	–	–
Perlroth, 2010 [38]	USA	Not reported	Multiple	77	38	–
			Multiple + AV	90	71	–
Halloran-Imperial/Pitt model, 2008 [39]	USA	Future pandemic strain	Multiple	73	–	–
			Multiple + AV	83	70	53
Halloran-UW/LANL model, 2008 [39]	USA	Future pandemic strain	Multiple	89	–	–
			Multiple + AV	94	92	86
Halloran-VBI model, 2008 [39]	USA	Future pandemic strain	Multiple	72	–	–
			Multiple + AV	91	81	64

*Abbreviations*: *ILI* influenza-like illness, *R*_*0*_ basic reproduction number, *Imperial/Pitt* Imperial College and the University of Pittsburgh, *UW/LANL* University of Washington and Fred Hutchinson Cancer Research Center in Seattle and the Los Alamos National Laboratories, *VBI* Virginia Bioinformatics Institute of the Virginia Polytechnical Institute and State University

^a^Percentage reduction = ((Attack rate in the absence of intervention – Attack rate with intervention) / Attack rate in the absence of intervention) × 100. Unless otherwise stated, percentage reduction applies to the intervention group in the epidemiological studies and to the general population in the modeling studies

^b^Single: Workplace social distancing (e.g., working from home, reduction in workplace contacts by 50%); Multiple: Workplace social distancing and other nonpharmaceutical interventions; AV: Antiviral treatment and prophylaxis; VAC: Vaccination

^c^Outcomes are surrogates for influenza: Rousculp - Attended work with severe ILI; Kumar - ILI; Lee - Seroconversion to 2009 influenza A(H1N1)

^d^Reduction in cumulative influenza attack rate in the workplace = 58%

^e^Reduction in cumulative influenza attack rate in the workplace = 81%

**Table 2:** Risk of bias in epidemiological studies of workplace social distancing, 2000–2017^*^

First author, year published	Outcome	Confounding	Selection	Intervention classification	Intervention deviations	Missing data	Outcome measurement	Reported results	Overall
Rousculp, 2010 [27]	Attend work with severe ILI	Moderate^a^	Low	Low	Low	Low	Moderate^b^	Serious^c^	Serious
Kumar, 2012 [28]	ILI	Serious^d^	Low	Moderate^e^	Low	Moderate^f^	Moderate^b^	Low	Critical
Lee, 2010 [29]	Seroconversion to 2009 influenza A(H1N1)	Serious^d^	Low	Low	Low	Low	Low	Low	Serious

*Abbreviations*: *ILI* influenza-like illness

*Assessed using the Risk of Bias in Epidemiological Studies of Interventions (ROBINS-I) tool. Risk of bias for each domain is classified into four categories: *low* (study is comparable to a well performed randomized trial), *moderate* (study is sound for a non-randomized study but cannot be considered comparable to a well performed randomized trial), *serious* (study has some important problems), and *critical* (study is too problematic to provide any useful evidence on the effects of intervention)

^a^A nonrandomized study is rarely at low risk of bias for confounding

^b^Subjective outcome self-reported by participants who were aware of the intervention group

^c^Results for attending work with ILI symptoms of any severity are not reported

^d^Inadequate or no adjustment

^e^Intervention status was determined retrospectively

^f^Response rate was 56%

Among the modeling studies, the most frequent workplace social distancing measure assessed was reduction in workplace contacts by 50% for the entire duration of the outbreak (Appendix). One study assessed the effect of extending the weekend. Several studies assessed the effect of combining workplace social distancing measures with other interventions, including school closure, community contact reduction, antiviral treatment and prophylaxis, and vaccination.

For studies modeling R_0_ ≤ 1.9, workplace social distancing measures alone (single intervention) showed a median reduction of 23% in the cumulative influenza attack rate in the general population (Table 1). Workplace social distancing measures combined with other nonpharmaceutical interventions showed a median reduction of 75% in the general population. Adding antiviral treatment and prophylaxis further reduced the influenza attack rate (median reduction = 90%) (Table 1). Subgroup analysis reported in two studies indicated that the percentage reduction was higher in workplaces than in the general population (Table 1 footnote).

The modeling studies reported that percentage reduction in cumulative influenza attack rate in the general population declined with higher R_0_ values (Table 1). The percentage reduction declined with increasing threshold for triggering interventions or with delayed implementation of interventions (Table 3). The percentage reduction also declined with lower compliance to workplace social distancing interventions (Table 4).

**Table 3:** Percentage reduction in cumulative influenza attack rate in the general population, by threshold for triggering intervention, modeling studies, 2000–2017

First author, year published	Intervention^a^	Threshold (%)^b^	Percentage reduction^c^		
			R_0_ ≤ 1.9	R_0_ = 2.0–2.4	R_0_ ≥ 2.5
Zhang, 2012 [31]	Single	0.02	18	–	–
		0.25	18	–	–
		1.5	18	–	–
		5.0	17	–	–
Halloran-Imperial/Pitt model, 2008 [39]	Multiple + AV	0.0001	99	96	64
		0.001	99	95	64
		0.01	99	94	64
		0.1	97	88	62
		1.0	83	70	53
		10.0	31	27	23
Halloran-UW/LANL model, 2008 [39]	Multiple + AV	0.0001	99	99	99
		0.001	99	99	99
		0.01	99	99	99
		0.1	99	99	98
		1.0	94	92	86
		10.0	57	54	47
Halloran-VBI model, 2008 [39]	Multiple + AV	0.0001	96	89	67
		0.001	96	89	67
		0.01	96	89	67
		0.1	96	88	66
		1.0	91	81	64
		10.0	55	49	50
Milne, 2008 [34, 40]	Single	Prior to first case	28	–	–
		2 weeks after 1st case	27	–	–
		4 weeks after 1st case	25	–	–
		6 weeks after 1st case	19	–	–
	Multiple	Prior to first case	94	–	95
		2 weeks after 1st case	94	–	89
		4 weeks after 1st case	86	–	29
		6 weeks after 1st case	73	–	1
Milne, 2013 [35]	Multiple	Immediately after 1st case	63	–	–
		2 weeks after 1st case	63	–	–
		4 weeks after 1st case	48	–	–

*Abbreviations*: *R*_*0*_ basic reproduction number, *Imperial/Pitt* Imperial College and the University of Pittsburgh, *UW/LANL* University of Washington and Fred Hutchinson Cancer Research Center in Seattle and the Los Alamos National Laboratories, *VBI* Virginia Bioinformatics Institute of the Virginia Polytechnical Institute and State University

^a^Single: Workplace social distancing; Multiple: Workplace social distancing and other nonpharmaceutical interventions; AV: Antiviral treatment and prophylaxis

^b^Threshold percent: Cumulative influenza illness attack rate in the general population that will trigger intervention

^c^Percentage reduction = ((Attack rate in the absence of intervention – Attack rate with intervention) / Attack rate in the absence of intervention) × 100

**Table 4:** Percentage reduction in cumulative influenza attack rate in the general population, by compliance with intervention, modeling studies, 2000–2017

First author, year published	Intervention^a^	Compliance (%)	Percentage reduction^b^		
			R_0_ ≤ 1.9	R_0_ = 2.0–2.4	R_0_ ≥ 2.5
Mao, 2011 [32]	Single	100	82	23	–
		90	61	20	–
		75	41	16	–
		50	22	9	–
Milne, 2008 [34]	Single	100	28	–	7
		90	26	–	7
		75	25	–	5
		50	17	–	2

*Abbreviation*: *R*_*0*_ basic reproduction number

^a^Single: Workplace social distancing

^b^Percentage reduction = ((Attack rate in the absence of intervention – Attack rate with intervention) / Attack rate in the absence of intervention) × 100

The percentage reduction in the peak daily attack rate was reported in five modeling studies (Table 5). These studies showed substantial effects in reducing the peak rate (median reduction for workplace social distancing alone = 45%). The time to influenza peak was reported in one epidemiological and four modeling studies (Table 6). These studies reported later peaks with intervention compared to no intervention (median delay to peak for workplace social distancing alone = 6 days).

**Table 5:** Percentage reduction in peak influenza attack rate in the general population, modeling studies, 2000–2017

First author, year published	Country	Influenza strain	Intervention^a^	Percentage reduction^b^		
				R_0_ ≤ 1.9	R_0_ = 2.0–2.4	R_0_ ≥ 2.5
Zhang, 2012 [31]	Singapore	Not reported	Single	28	–	–
Mao, 2011 [32]	USA	Seasonal scenario (R_0_ = 1.4) and a pandemic scenario (R_0_ = 2.0)	Single	97	53	–
Xia, 2013 [33]	China	2009 Influenza A(H1N1) pandemic	Single	51	–	–
			Single + VAC	91	–	–
Milne, 2008 [34]	Australia	Pandemic strain	Single	39	25	18
			Multiple	97	99	99
Milne, 2013 [35]	Papua New Guinea	Pandemic strain	Multiple	91	–	–

*Abbreviations*: *R*_*0*_ basic reproduction number, *VAC* vaccination

^a^Single: Workplace social distancing; Multiple: Workplace social distancing and other nonpharmaceutical interventions; VAC: Vaccination

^b^Percentage reduction = ((Attack rate in the absence of intervention – Attack rate with intervention) / Attack rate in the absence of intervention) × 100

**Table 6:** Time to influenza peak, epidemiological and modeling studies, 2000–2017^a^

First author, year published	Country	Influenza strain	Intervention^b^	Days to peak		
				R_0_ ≤ 1.9	R_0_ = 2.0–2.4	R_0_ ≥ 2.5
Lee, 2010 [29]	Singapore	2009 influenza A(H1N1) pandemic	Multiple	Peak later (unspecified) with intervention	–	–
Zhang, 2012 [31]	Singapore	Not reported	Single	Peak 1 day later with intervention	–	–
Mao, 2011 [32]	USA	Seasonal scenario (R_0_ = 1.4) and a pandemic scenario (R_0_ = 2.0)	Single	Peak 89 days later with intervention	Peak 18 days later with intervention	–
Xia, 2013 [33]	China	2009 influenza A(H1N1) pandemic	Single	Peak 6 days later with intervention	–	–
Milne, 2013 [35]	Papua New Guinea	Pandemic strain	Multiple	Peak 13 days later with intervention	–	–

*Abbreviations*: *R*_*0*_ basic reproduction number

^a^For the modeling studies (Zhang [31], Mao [32], Xia [33], Milne [35]), time to influenza peak is reported for the general population

^b^Single: Workplace social distancing; Multiple: Workplace social distancing and other nonpharmaceutical interventions

## Discussion

Epidemiological and modeling studies indicated that workplace social distancing reduced the overall number of influenza cases. It also reduced and delayed the influenza peak. The modeling studies reported that the reduction in influenza cases was more pronounced when workplace social distancing was combined with other interventions. However, the effectiveness was estimated to decline with higher R_0_ values, delayed triggering of workplace social distancing, or lower compliance.

Droplets, and possibly aerosols, generated by coughs and sneezes are a major source of influenza transmission [42–45]. Social distancing in workplaces can decrease the risk of person-to-person influenza transmission by reducing droplet transmission that occurs within 3–6 ft [43]. Workplace social distancing and other nonpharmaceutical or pharmaceutical interventions implemented together can act in complementary ways to reduce virus transmission [3, 46]. Social distancing was estimated to be less effective for higher R_0_ values. The lower effectiveness could be because social distancing may be less likely to reduce the effective reproduction number to below one if R_0_ is higher [6]. The lower effectiveness with delayed triggering or lower compliance may be due to several factors. Delayed triggering of workplace social distancing precludes the opportunity to impact cases that have already occurred and represents a missed opportunity to diminish further transmission. Lower compliance increases the opportunity for person-to-person transmission.

This systematic review has several potential limitations. First, most of the included studies were based on modeling and few were in actual settings. Models can fill gaps when decisions must be made when there is a paucity of information [47]. However, more epidemiological studies are needed on social distancing in actual settings. Second, we did not assess the quality of the modeling studies. Input parameters used in simulation models include the population characteristics that describe exposure points (e.g., households, schools, workplaces); the population’s behaviors that represent exposure frequencies (e.g., contact rates and durations); and disease transmission parameters [15, 48]. There are few empirical studies on contact rates at workplaces [30]. No studies provided empirical information regarding the impact of workplace social distancing measures on changing workplace contact rates. Third, the studies included did not report the effects of workplace social distancing on two of our primary outcomes of interest (lost workdays, harms). The impact on lost workdays would represent the balance between potential work loss associated with social distancing (which can be mitigated by the ability to work from home) and sick days averted by reduction in influenza transmission and illness. One study reported that a lower proportion of Hispanic and African American workers than of white workers are able to work at home [28]. This observation indicates the need to consider the potential for racial or ethnic disparities. Fourth, because the effectiveness of workplace social distancing would depend on many factors, including R_0_, timing of implementation, and compliance, it is difficult to estimate the likely magnitude of impact in a future pandemic. Finally, only one of the included studies represented a lower-income country setting [35]. The findings of our evidence synthesis may not be generalizable to lower-income countries that differ in demography and contact patterns.

There were several strengths. We conducted a comprehensive search of the literature that focused on workplace social distancing. The studies included in our review assessed the effect of workplace social distancing measures alone or combined with other interventions, allowing assessment of the relative effectiveness of single and combination interventions.

To our knowledge, our systematic review is the first one that focuses on workplace social distancing. A previous systematic review of modeling studies published during 1990 to 2009 assessed the effectiveness of pharmaceutical (vaccines and antiviral agents) and nonpharmaceutical (case isolation, quarantine, personal hygiene measures, social distancing, and travel restrictions) strategies for pandemic influenza response [49]. This previous review, which was based on 19 articles (five of these articles included workplace closure and two included workplace social distancing), concluded that combination strategies increased the effectiveness of individual strategies. Other systematic reviews that have assessed the effectiveness of interventions in reducing pandemic influenza transmission did not examine workplace social distancing measures [50].

An increasing trend in the ability to telework aligns with recommendations for social distancing in a pandemic, but teleworking is less feasible for many occupations [51]. About 24% of employed persons did some or all of their work at home in 2015, ranging from about 35% in managerial and professional occupations to only 6% in production occupations. It is estimated that it is possible for 50% of the US workforce to telework at least partially [52]. Studies that examine feasible and acceptable workplace social distancing strategies in a variety of work settings are needed to improve pandemic preparedness. Because contact patterns differ in different types of industries and workplaces, the impact of social distancing in various settings needs to be assessed. Further research is needed to facilitate development of higher-fidelity models of influenza transmission in the workplace for model-based evaluation of NPI effects in different industries. Cross-sectional epidemiological studies can be used to assess the prevalence of workplace social distancing measures, but this design is not optimal to assess effectiveness because of inherent biases [53]. Because randomizing employers or employees to social distancing or control arms may not be feasible, prospective cohort studies may provide the best available evidence on effectiveness. Employers that have implemented workplace social distancing measures could be compared to those that have not, particularly during a pandemic. However, attention should be paid to collecting data on potential confounding variables and using outcomes that are defined in an objective manner (e.g., laboratory-confirmed influenza illness, sensitive and specific case definitions using electronic medical records data).

Our findings have several implications. First, the effectiveness of workplace social distancing was estimated to decline with higher R_0_ values. This finding has ramifications regarding the intensity of community mitigation measures that may be needed in atypical pandemics with higher R_0_ values. Second, effectiveness declined with delayed triggering. The ability of local surveillance systems to accurately detect influenza circulation in the community to inform triggering decisions will depend on several factors, including the sensitivity and specificity of the case definition (laboratory testing of all ill patients may not be possible), the representativeness of the reported cases, and the completeness of reporting. Algorithms for estimating the total number of cases in a community based on cases detected by local surveillance systems, or use of proxy measures, may be needed to guide triggering decisions. Decision-makers should weigh the benefits versus disruptions of implementing workplace social distancing measures in the context of pandemic severity [46]. Third, effectiveness declined with lower compliance. Triggering social distancing too early can contribute to lower compliance because of intervention fatigue [54]. Finally, effectiveness was reported to be greater when workplace social distancing was combined with other nonpharmaceutical or pharmaceutical interventions. The findings underscore the importance for coordination between employers and state/local health departments to potentially enhance impact using a combination of measures.

## Conclusions

Our systematic review shows that there are few epidemiological studies in actual settings. More research is needed to assess the effectiveness of social distancing measures in a variety of industries and work settings. The included epidemiological and modeling studies indicate that social distancing in non-healthcare workplaces reduces the overall as well as the peak number of influenza cases. It also delays the influenza peak. The finding that reduction in influenza cases is more pronounced when workplace social distancing is combined with other nonpharmaceutical or pharmaceutical interventions highlights the importance of using a combination of measures to reduce the transmission of pandemic influenza.

### Additional files

Additional file 1: **Table S1.** PRISMA 2009 checklist. (DOC 63 kb)

Additional file 2: **Table S2.** Data for percentage reduction in cumulative influenza attack rate. (XLSX 13 kb)

Additional file 3: **Table S3.** Data for percentage reduction in cumulative influenza attack rate in the general population, by threshold for triggering intervention. (XLSX 14 kb)

Additional file 4: **Table S4.** Data for percentage reduction in cumulative influenza attack rate in the general population, by compliance with intervention. (XLSX 11 kb)

Additional file 5: **Table S5.** Data for percentage reduction in peak influenza attack rate in the general population. (XLSX 11 kb)

Additional file 6: **Table S6.** Data for time to influenza peak. (XLSX 9 kb)

## Appendix

**Table 7:** Description of studies included in a review of effectiveness of workplace social distancing to reduce influenza transmission, 2000–2017

First author, year published	Study design	Influenza strain and transmissibility (R_0_)	Population, setting, and number of people (n)	Intervention(s) and comparator^a^	Relevant outcomes
Epidemiological studies					
Rousculp, 2010 [27]	Cohort (participants surveyed at baseline and monthly from October 2007 to April 2008)	Seasonal influenza A(H3N2), 2007–2008	Employees of three large US companies (national retail chain, transportation, and manufacturing) (*n* = 793 employees with ILI)	Single: Can work from home Comparator: Cannot work from home	Attended work for ≥1 day while ILI symptoms were most severe (which would result in transmission to co-workers)
Kumar, 2012 [28]	Cross-sectional (survey completed from January 22 to February 1, 2010)	2009 influenza A(H1N1) pandemic	Random sample of US adults from the Knowledge Networks online research panel (*n* = 2079)	Single: Can work from home Comparator: Cannot work from home	ILI during April 2009 to date survey completed
Lee, 2010 [29]	Cohort (June 22 to October 9, 2009)	2009 influenza A(H1N1) pandemic	Singapore military personnel (*n* = 907)	Multiple: Standard pandemic plan (provided general health education on respiratory and hand hygiene and advised to seek medical care if ill) + segregation of units into subgroups as small as 20 individuals (including having different activity and meal times, and times of entry and exit from camp) + daily temperature and symptom monitoring with provision of home medical leave Comparator: Standard pandemic plan	Seroconversion to 2009 influenza A(H1N1); time to peak (based on onset of symptoms among those who seroconverted)
Modeling studies					
Timpka, 2016 [30]	Model based on an ontology system. Mean duration of outbreak in the reference model = 92 days	Future pandemic strain	General population in Linkoping municipality, Sweden (*n* = 136,000)	Single: Social distancing that decreases workplace influenza virus transmission probability by 50% Comparator: No intervention	Cumulative influenza attack rate
Zhang, 2012 [31]	Agent-based model. Simulated for 200 days. Intervention is triggered at a threshold of 0.02%	R_0_ = 1.9	General population in Singapore (*n* = 480,000, representing a 10% sample)	Single: Team-based rotational workforce shift for 6 weeks (i.e., each company or institution splits its employees into two work teams and minimizes contacts between the teams through 7-day rotations of staying at home or attending work) Comparator: No intervention	Cumulative and peak influenza attack rates; peak attack day
Mao, 2011 [32]	Agent-based model. Simulated for 200 days. Intervention is triggered at a threshold of 0.1%	Seasonal scenario (R_0_ = 1.4) and a pandemic scenario (R_0_ = 2.0)	General population in Buffalo, New York, USA (*n* = 985,001)	Single: Weekend extension by 3 days (Sat, Sun, Mon, Tues, Wed). Comparator: No weekend extension (Sat, Sun)	Cumulative influenza attack rate; daily new cases
Xia, 2013 [33]	Compartmental model. Simulated for 200 days	2009 influenza A(H1N1) pandemic. R_0_ = 1.5	General population in Hong Kong, China (*n* = 7 million)	Single: Workplace contact reduction Single + Vaccination: Workplace contact reduction + vaccination with a coverage of 2.5% Comparator: No intervention	Peak infectious population; days to peak
Milne, 2008 [34, 40]	Agent-based model. Intervention is triggered before the introduction of the first infected case	Pandemic strain. R_0_ = 1.5, 2.0, 2.5	General population in Albany, Australia (*n* = 30,000)	Single: Workplace nonattendance (each person attending a workplace has a 50% chance each day of staying home instead of attending the workplace) Multiple: Workplace nonattendance + school closure + case isolation + community contact reduction Comparator: No intervention	Cumulative and peak influenza attack rates
Milne, 2013 [35]	Agent-based model. Intervention is triggered 2 weeks after the first case	Pandemic strain. R_0_ = 1.88	General population in Madang, Papua New Guinea (*n* = 35,000)	Multiple: Workplace nonattendance (each person attending a workplace has a 50% chance each day of staying home instead of attending the workplace) + community contact reduction (50%) + school closure Comparator: No intervention	Cumulative influenza attack rate; daily incident cases
Miller, 2008 [36]	Discrete event model. Intervention is triggered after the first infection in city	Influenza A(H3N2) in population with no prior immunity	General population in San Antonio, Texas, USA (*n* = 1.4 million)	Multiple: Reducing the number of community contacts to 50% by day 21 after first infection (representing closing schools and churches, banning public gatherings, and encouraging people to work from home) Comparator: No intervention	Cumulative influenza attack rate
Andradottir, 2011 [37]	Individual-based compartmental model. Simulated for 180 days. Intervention is triggered at a threshold of 0.01%	2009 influenza A(H1N1) pandemic. R_0_ = 1.4	General population in Hamilton, Canada (*n* = 649,565)	Multiple: Social distancing (20% reduction in workplace and general community contacts) + school closure Multiple + Vaccination: Social distancing + school closure + vaccination of up to 35% of the population with a vaccine with 40% vaccine efficacy (60-day delay in vaccination after trigger threshold is reached) Multiple + Antiviral: Social distancing + school closure + antiviral treatment and prophylaxis of up to 10% of the population Comparator: No intervention	Cumulative influenza attack rate
Perlroth, 2010 [38]	Agent-based model. Intervention is triggered at a threshold of 0.1%	R_0_ = 1.6, 2.1	General population in a small US town (*n* = 10,000)	Multiple: Adult social distancing (work contacts reduced by 50%) + child social distancing + school closure Multiple + Antiviral: Adult social distancing + child social distancing + school closure + antiviral treatment and prophylaxis Comparator: No intervention	Cumulative influenza attack rate
Halloran (Imperial-Pitt model), 2008 [39]	Agent-based model. Results at day 180 of the epidemic are reported. Intervention is triggered at a threshold of 1%	Future pandemic strain. R_0_ = 1.9, 2.4, 3.0	General population in Chicago, Illinois, USA (*n* = 8.6 million)	Multiple + Antiviral: Workplace social distancing (workplace contacts reduced by 50%) + community social distancing + school closure + interventions within the households of ascertained influenza cases (antiviral treatment and prophylaxis, home isolation of cases, and quarantine of household contacts) Multiple: Above, excluding antiviral treatment and prophylaxis Comparator: No intervention	Cumulative influenza attack rate
Halloran (UW/LANL model), 2008 [39]	Agent-based model. Results at day 180 of the epidemic are reported. Intervention is triggered at a threshold of 1%	Future pandemic strain. R_0_ = 2.1, 2.4, 3.0	General population in Chicago, USA (*n* = 8.6 million)	Multiple + Antiviral: Workplace social distancing (workplace contacts reduced by 50%) + community social distancing + school closure + interventions within the households of ascertained influenza cases (antiviral treatment and prophylaxis, home isolation of cases, and quarantine of household contacts) Multiple: Above, excluding antiviral treatment and prophylaxis Comparator: No intervention	Cumulative influenza attack rate
Halloran (VBI model), 2008 [39]	Agent-based model. Results at day 180 of the epidemic are reported. Intervention is triggered at a threshold of 1%	Future pandemic strain. R_0_ = 2.1, 2.4, 3.0	General population in Chicago, USA (*n* = 8.6 million)	Multiple + Antiviral: Workplace social distancing (workplace contacts reduced by 50%) + community social distancing + school closure + interventions within the households of ascertained influenza cases (antiviral treatment and prophylaxis, home isolation of cases, and quarantine of household contacts) Multiple: Above, excluding antiviral treatment and prophylaxis Comparator: No intervention	Cumulative influenza attack rate

*Abbreviations*: *ILI* influenza-like illness, *R*_*0*_ basic reproduction number, *Imperial/Pitt* Imperial College and the University of Pittsburgh, *UW/LANL* University of Washington and Fred Hutchinson Cancer Research Center in Seattle and the Los Alamos National Laboratories, *VBI* Virginia Bioinformatics Institute of the Virginia Polytechnical Institute and State University

^a^Unless otherwise stated, the modeling studies assumed that the duration of social distancing in workplaces was for the entire influenza outbreak period

## Abbreviations

AV: Antiviral treatment and prophylaxis
CINAHL: Cumulative Index to Nursing and Allied Health Literature
ILI: Influenza-like illness
Imperial/Pitt: Imperial College and the University of Pittsburgh
NPI: Nonpharmaceutical intervention
PRISMA: Preferred Reporting Items for Systematic Reviews and Meta-Analysis
R_0_: Basic reproduction number
US: United States
UW/LANL: University of Washington and Fred Hutchinson Cancer Research Center in Seattle and the Los Alamos National Laboratories
VAC: Vaccination
VBI: Virginia Bioinformatics Institute of the Virginia Polytechnical Institute and State University
